# Differences in Multidisciplinary and Interdisciplinary Treatment Programs for Fibromyalgia: A Mapping Review

**DOI:** 10.1155/2017/7261468

**Published:** 2017-05-23

**Authors:** Emanuele Maria Giusti, Gianluca Castelnuovo, Enrico Molinari

**Affiliations:** ^1^Department of Psychology, Catholic University of Milan, Milan, Italy; ^2^Istituto Auxologico Italiano IRCCS, Psychology Research Laboratory, San Giuseppe Hospital, Verbania, Italy

## Abstract

Fibromyalgia is a multifaceted chronic pain syndrome and the integration of different health disciplines is strongly recommended for its care. The interventions based on this principle are very heterogeneous and the difference across their structures has not been extensively studied, leading to incorrect conclusions when their outcomes are pooled. The objective of this mapping review was to summarize the characteristics of these programs, with particular focus on the integration of their components. We performed a search of the literature about treatments for fibromyalgia involving multiple disciplines on PubMed and Scopus. Starting from 560 articles, we included 22 noncontrolled studies, 10 controlled studies, and 17 RCTs evaluating the effects of 38 multidisciplinary or interdisciplinary interventions. The average quality of the studies was low. Their outcomes were usually pain intensity, quality of life, and psychological variables. We created a map of the programs based on the degree of integration of the included disciplines, which ranged from a juxtaposition of few components to a complex harmonization of different perspectives obtained through teamwork strategies. The rehabilitation programs were then thoroughly described with regard to the duration, setting, therapeutic components, and professionals included. The implications for future quantitative reviews are discussed.

## 1. Introduction

Fibromyalgia is a chronic pain condition of unknown etiology which affects mainly women and is characterized by stiffness, fatigue, disturbed sleep, cognitive impairment, and psychological distress, posing a significant threat to the quality of life of affected individuals [1–3]. The complex features of the syndrome and its various symptoms are partially explained by central sensitization processes that interact with psychological and social factors, leading to a phenomenon where the impact of each component is multiplied and acts in a synergic manner [4, 5]. Given its multifaceted nature and the poor efficacy of standard medical interventions, the integration of different health disciplines for its understanding and for the development of specific treatments has long been advocated [6–10]. As a result, a large number of interventions combining techniques drawn from different fields (e.g., medicine, psychology, and physical therapy) have been developed and have proven to be effective for the improvement of the various symptoms of the syndrome [11–15]. Nonetheless, these programs are very heterogeneous in terms of duration, objectives, setting, format, therapeutic components, and professionals involved. This great variability is reflected by different organizational frameworks of the pain treatment facilities and casts some doubt on the possibility of pooling the results of trials evaluating their interventions [16, 17]. The composition of the rehabilitation teams and the integration between their members seem to be especially important in distinguishing the various programs, since there is a wide difference with regard to how their various disciplines are harmonized, combined, or juxtaposed. Conversely, terms such as “multimodal,” “multicomponent,” “multidisciplinary,” and “interdisciplinary” are often used as synonyms [18, 19]. From a theoretical point of view, the difference between these concepts is substantial. The terms “multimodal” and “multicomponent” have not received yet a clear definition. Multimodality generally refers to the combination of multiple therapeutic components, not necessarily provided by different operators. The expression “multicomponent treatments” indicates the presence of interventions provided by different team members, without clarifying how they are integrated [20]. Conversely, the other two terms define how these components are combined. Multidisciplinarity refers to the addition of the competencies of multiple professionals who stay within the boundaries of their fields, whereas interdisciplinarity denotes that the various disciplines are coordinated toward a common and coherent approach [19]. Multidisciplinary and interdisciplinary treatments are therefore conceptually different and this difference should be reflected in their structures. Especially in the case of programs addressing a complex disorder, such as the fibromyalgia syndrome, this issue must be taken into account for the sake of a critical appraisal of the literature, which can be a preliminary step for quantitative synthesis. The objective of this review of the literature is therefore to describe, map, and summarize the characteristics of treatments for fibromyalgia involving multiple health disciplines, with a focus on how these disciplines are integrated.

## 2. Methods

A mapping review of the literature was performed. Mapping reviews usually aim to retrieve, catalog, and map all the evidence about a research topic [21, 22]. We adapted their methodological design in order to use rehabilitation programs as units of analysis. Therefore, instead of investigating the characteristics and the results of the included papers, we focused on the information about the interventions that were described.

We conducted a comprehensive search in PubMed and Scopus of all the available original research reports describing and evaluating multidisciplinary, interdisciplinary, or multicomponent interventions for fibromyalgia up to April 2016. We used these keywords in both databases: fibromyalgia, multidisciplinary, interdisciplinary, multicomponent, and integrated treatments. The studies were scanned according to the inclusion (description and evaluation of a multicomponent intervention involving at least two operators from different disciplines, fibromyalgia as primary diagnosis, and English language) and exclusion (review as publication type, single-case studies, and workplace interventions) criteria. Since our aim was to describe the various treatment programs and we were not interested in a quantitative evaluation of their outcomes, we included both RCTs, nonrandomized trials and noncontrolled trials. We also included studies evaluating outcome predictors, if they provided data about the effects of the interventions. A first scanning was based only on the titles and the abstracts of the retrieved research articles. After this first step, the full text of the remaining papers was accessed and we assessed the methodological quality of the studies. Since we included both controlled and noncontrolled trials, we adapted the SIGN checklist for randomized controlled trials [23] adding four items from the Downs and Black Checklist [24] (i.e., use of appropriate analyses, assessment of confounders, use of a representative sample, and intervention integrity) (Table 1). We used its scores to rate the studies as of “high,” “medium,” “low,” and “insufficient” quality. Questions from 2 to 6 were not considered during the evaluation of noncontrolled studies.

We then employed a specifically created data extraction sheet to retrieve all the available information about number of study participants, dropout, professionals involved in the treatment, group/individual setting, inpatient/outpatient format, duration, components of the interventions, and treatment outcomes. When possible, we tried to locate and report the information about how the different disciplines are integrated with each other, in order to provide a brief outline of the program. In case of unclear or missing information, we initially tried to locate other descriptions consulting other studies of the same authors. If no other relevant studies were found, we tried to contact the authors of the articles and we asked them for additional information. All the data about the treatments were finally inserted in a MS Excel spreadsheet and were used to map the included interventions.

## 3. Results

### 3.1. Study Selection and Characteristics

**Figure 1:**
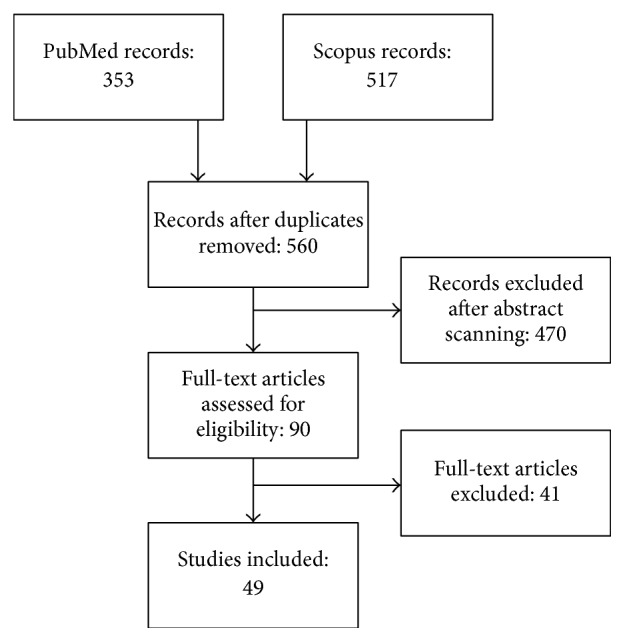
PRISMA flow chart diagram for study selection.

The initial database search identified 560 records, of which 90 were selected for a full-text examination. The final database included 49 papers describing 38 different interventions that were administered to a total of 6013 subjects (Figure 1).

We found 22 papers describing noncontrolled studies, 10 describing controlled studies, and 17 describing RCTs (Table 2). The majority of the articles mainly reported the effects of a multicomponent treatment, whereas six papers mainly reported analyses on outcome predictors, one trial compared two multidisciplinary treatments, and three trials evaluated the effects of adding a treatment component to existing multidisciplinary treatments. The average methodological quality of the studies was low; 3 noncontrolled trials, 5 controlled trials, and 10 RCTs were rated as of medium quality and 6 studies were rated as of high quality. Their outcomes were usually pain intensity, psychological variables (such as anxiety, depression, distress, pain catastrophizing, coping skills, and self-efficacy), quality of life, and improvement in the overall score of the Fibromyalgia Impact Questionnaire.

### 3.2. Multidisciplinary and Interdisciplinary Programs

**Figure 2:**
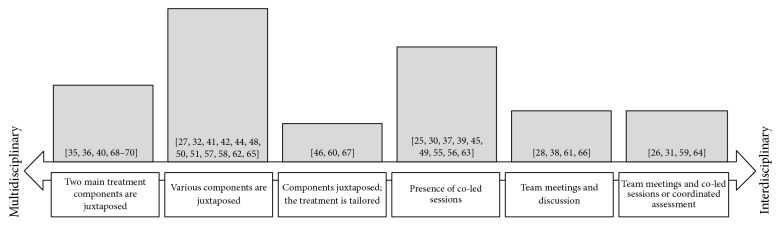
Map of the rehabilitation programs.* Note*. The height of the bars is proportional to the frequency of the interventions included in the relative category. The references of the studies describing these interventions are in brackets.

Among the studies included in the review, the terms “multimodal” and “multicomponent” were rarely employed and were never used as primary descriptors of the structure of the interventions, whereas 25 treatments were defined as “multidisciplinary” and 11 as “interdisciplinary.” However, there was not a clear distinction between these concepts. In some cases, the terms were used interchangeably or were incorrectly employed (e.g., [71, 73] or [62]). More generally, the programs were very heterogeneous with regard to the strategies that were employed to integrate the different perspectives of the operators and can be mapped in a continuum which starts with multidisciplinary treatments involving few disciplines whose components are simply added and ends with more complex interventions that are based on a coordinated assessment and care (see Figure 2).

Most of the treatments were based on various components that were juxtaposed, so that the patient met the different health operators in different moments of the program in a standardized manner. A few interventions were more flexible and allowed the operators to tailor the programs, in terms of number and intensity of the therapeutic components, to the needs and characteristics of the patients. More integrated approaches were organized so that some or the majority of the treatment sessions were led by two or more professionals jointly. In other cases, weekly team meetings were planned to discuss the patients, allowing a constant dialog between the operators. This could be added to other strategies, such as providing education about interdisciplinary work or tailoring the various components to the patients, in order to provide more effective integration of the different disciplines.

### 3.3. Description of the Interventions

The main characteristics of the interventions are reported in Table 3. Most of the programs were based on an outpatient setting; only five intensive treatments were provided in hospital inpatient services. Only four programs were mainly administered individually, six interventions included both group and individual activities, and the other 28 treatments were mainly group-based. There was a great variety with regard to the duration and the intensity of the interventions. The median duration was 7 weeks, but there were both very brief treatments lasting for less than a week (e.g., [70]) and very long treatments lasting for one year (e.g., [27]). With regard to the intensity, there were both intensive programs (e.g., [28]) and interventions including less than twelve hours of therapies (e.g., [70]), with a median of 42 hours of treatment.

The number and the choice of the disciplines that were integrated in the various interventions were very variable, as well as their relative importance. As a consequence, it was not possible to categorize the programs creating clusters based on the professionals included. Physicians, physical therapists, and psychologists were the most employed operators, followed by occupational therapists, nurses, social workers, dietitians, and other professionals such as Qi-gong instructors, kinesiologists, and massage therapists.

The main therapeutic components included in the various rehabilitation programs were education about fibromyalgia and physical exercise, which were present in 36 out of 38 programs. Education was generally based on group lectures about the syndrome, its symptoms, its prognosis, and the biopsychosocial factors influencing its long-term progression and its daily course. The prescribed physical activity was various and included aerobic, pool, and strengthening exercises. 19 programs provided some psychotherapy sessions under the cognitive-behavioral approach, generally focused on symptom management. Both the educational lectures and the psychotherapeutic sessions could also be focused on teaching the patients to self-manage their symptoms and on enhancing group discussions among the participants. Besides these common therapeutic components, other forms of interventions were usually provided. These include, for instance, occupational therapies, biofeedback, massage therapies, relaxation, Acceptance and Commitment Therapy sessions, and dietary counseling.

## 4. Discussion

The objective of this review was to describe the characteristics of the available integrated interventions for fibromyalgia, focusing on how the different disciplines are integrated with each other. Our study, therefore, is embedded in two lines of inquiry, that is, the discussion about multidisciplinarity and interdisciplinarity in the healthcare and the evaluation of multicomponent treatment for chronic pain conditions.

With regard to the first, our study is in line with and finds support for the predominant theoretical perspective which describes multidisciplinarity and interdisciplinarity as two parts of the same continuum [18, 19]. Employing the original perspective of analyzing how the integration of the different disciplines is provided by the various programs, we found that the possible organizational frameworks are multiple and are characterized by different degrees of complexity. On the one hand, less integrated programs provide a juxtaposition of two or more different disciplines, which are simply added. These programs can be seen as the prototype of multidisciplinary interventions, since every operator works independently, there is no overlap between the different treatment modalities, and the program is generally organized in a vertical manner [75]. However, we found that there are multidisciplinary programs which use a more flexible and integrated framework, which allows us to modify the treatment and to involve the patient in the decisions about his/her care. In more cohesive programs, some or the majority of the education or pain management sessions are jointly conducted by different operators, allowing the professionals to build a shared perspective regarding the various topics. Prototypical interdisciplinary models seem however to be based on weekly team discussions about the patients, which can be accompanied by other strategies, such as education about interdisciplinary work, a coordinated assessment, or providing sessions jointly conducted by the operators. The presence of team meetings seems to be considered by the authors of the studies included in this review as the main characteristic which defines their treatment as “interdisciplinary,” partly overlooking other components such as creating common goals, making team conferences with the patient and his/her family, and providing shared leadership and a comprehensive assessment [75, 76].

With regard to the evaluation of the multicomponent interventions for fibromyalgia, our study represents a first step for conducting a quantitative synthesis of the literature. As we had hypothesized, we found that the programs included in our review were very heterogeneous in terms of number, type, and integration of the components and professionals, as well as in terms of duration and intensity. Previous analyses of the literature did not successfully address this diversity. Hauser and colleagues [20] performed a meta-analysis on 7 RCTs evaluating multicomponent treatments for fibromyalgia, finding support for their short-term efficacy. The author conducted a sensitivity analysis based only on the methodologic quality and the duration of the programs. A previous Cochrane review [77] attempted to include all the interventions which contained both a medical and a psychological, social, or vocational component, finding four RCTs and showing that these interventions did not provide quantifiable benefits. Different from our study, the authors did not address the differences with regard to format, setting of the program, and integration of operators and therapeutic components. More recently, Papadopoulou et al. [11] evaluated all the pharmacological and nonpharmacological interventions for fibromyalgia, finding support for multidisciplinary treatments based on 8 RCTs, which were not described. Similarly, other reviews focusing on various chronic pain conditions found that multicomponent programs are effective for the treatment of fibromyalgia but did not address their heterogeneity [12, 78]. Our study was not aimed at a quantitative evaluation of the outcomes of these programs but provides a detailed description of the various programs identifying some key characteristics that must be taken into account by future systematic reviews and meta-analyses. In particular, the most varying factors were the duration and the intensity of the programs, the treatment components, and the strategies of integration of the operators. It is needed that future quantitative reviews separately analyze studies evaluating treatments with different structures and assess the moderator effect of the temporal characteristics of the programs.

Some important remarks need to be made. On the one hand, we focused only on studies which included multiple disciplines performed by multiple operators. Therefore, we excluded the papers which did not state clearly that multiple professionals were present and we focused on programs performed by rehabilitation teams. As a consequence, our description is limited to these treatments and it is possible that some multidisciplinary programs were excluded due to poor descriptions of their structure. In addition, our findings are based on brief reports included in scientific papers, and it is possible that important characteristics of the evaluated interventions were missing and that the whole of the multidisciplinary and interdisciplinary rehabilitation programs is misrepresented by our sample. Finally, our electronic search was limited to two databases and to papers written in English language, limiting the comprehensiveness of the review.

However, to our knowledge, this is the first study which attempts to map the characteristics of multicomponent interventions for fibromyalgia. In addition, performing this mapping review allowed us to create a comprehensive database of studies that can be used for further analyses and that is available upon request. We are persuaded that the integration of the various disciplines is a key factor for the treatment of such a condition. It is possible that the more the perspectives of the various professionals are integrated, the more the patients are able to comprehend the complexity of their syndrome and can be conscious about how the different biological, psychological, and social factors affect its course.

## 5. Conclusions

Multidisciplinary and interdisciplinary treatments must be considered as two ends of the same continuum. The degrees of integration of their component do not depend only on the juxtaposition or on the overlap between the various competences but rely on the possibility of tailoring the programs to the patients or on the presence of co-led sessions, team meetings, and a coordinate assessment. The various studies are very heterogeneous with regard to most of the variables described in our review. This diversity will need to be taken into account by future quantitative reviews of the literature.

## Figures and Tables

**Table 1:** Adapted SIGN checklist.

(1)	The study addresses an appropriate and clearly focused question.
(2)	The assignment of subjects to treatment groups is randomized.
(3)	An adequate concealment method is used.
(4)	The design keeps subjects and investigators “blind” about treatment allocation.
(5)	The treatment and control groups are similar at the start of the trial.
(6)	The only difference between groups is the treatment under investigation.
(7)	All relevant outcomes are measured in a standard, valid, and reliable way.
(8)	Were the statistical tests used to assess the main outcomes appropriate?
(9)	Were those subjects who were prepared to participate representative of the entire population from which they were recruited?
(10)	What percentage of the individuals recruited into each treatment arm of the study dropped out before the study was completed?
(11)	Are all the subjects analyzed in the groups to which they were randomly allocated (application of an intention-to-treat analysis)?
(12)	Where the study is carried out at more than one site, results are comparable for all sites.
(13)	Are the distributions of principal confounders in each group of subjects to be compared clearly described?
(14)	How well was the study done to minimize bias?
(15)	Is the overall effect due to the study intervention?

*Note*. During the assessment of noncontrolled studies, items from (2) to (6) were not considered.

**Table 2:** Description of the included studies.

Reference	Country	Design	Type of study	Comparison group	Subjects (dropout)	Outcomes	Overall quality
Åkerblom et al., 2015 [25]	Sweden	Noncontrolled study	Outcome predictors	N/A	409 (145)	Pain, psychological factors, pain interference	Medium
Amris et al., 2014 [26]	Netherlands	RCT	Comparison with control	WL	191 (21)	QOL, pain, psychological factors, FIQ, movement	High
Anderson and Winkler, 2006 [27]	US	Controlled trial	Comparison with control	TAU	98 (5)	QOL, pain, psychological factors, FIQ, tender points	Low
Angst et al., 2006 [28]	Switzerland	Noncontrolled study	Comparison with control	TAU	225 (100)	QOL, pain, pain interference, psychological factors	Low
Angst et al., 2009 [29]		Controlled trial	Pre-post evaluation	TAU	331 (121)	QOL, pain, psychological factors	Low
Bailey et al., 1999 [30]	Canada	Noncontrolled study	Pre-post evaluation	N/A	149 (43)	Pain, pain interference, psychological factors, FIQ, physical fitness	Low
Bourgault et al., 2015 [31]	Canada	RCT	Comparison with control	WL	58 (15)	QOL, pain, psychological factors, pain interference, FIQ, perception of improvement	Medium
Carbonell-Baeza et al., 2011 [32]	Spain	Controlled trial	Comparison with control	TAU + education	75 (10)	Tender points count, pain threshold, physical fitness, BMI	Medium
Carbonell-Baeza et al., 2011 [33]		Controlled trial	Comparison with control	TAU + education	75 (10)	QOL, pain, psychological factors, FIQ	Medium
Carbonell-Baeza et al., 2012 [34]		Controlled trial	Comparison with additional treatment	Biodanza	38 (7)	QOL, pain, psychological factors, FIQ, physical fitness, tender points	Medium
Casanueva-Fernández et al., 2012 [35]	Spain	RCT	Comparison with control	TAU + education	34 (6)	QOL, pain, psychological factors, FIQ, fatigue, sleep, tender points count, pain threshold, physical fitness	Medium
Castel et al., 2013 [36]	Spain	RCT	Comparison with control	TAU	155 (28)	Pain, psychological factors, FIQ, sleep	High
Cedraschi et al., 2004 [37]	Switzerland	RCT	Comparison with control	WL	164 (35)	QOL, pain, psychological factors, FIQ, tender points count, satisfaction	High
De Rooij et al., 2013 [38]	Netherlands	Noncontrolled study	Outcome predictors	N/A	138 (18)	Pain, psychological factors, pain interference, perception of improvement	Low
Gustafsson et al., 2002 [39]	Sweden	Controlled trial	Comparison with control	TAU	43 (2)	Movement, pain drawings, pain, QOL, pain interference	Low
Hammond and Freeman, 2006 [40]	UK	RCT	Comparison with control	Relaxation training	183 (80)	FIQ, psychological factors, physical activity, healthcare use, perception of improvement	Medium
Hamnes et al., 2012 [41]	Norway	RCT	Comparison with control	WL	147 (29)	Psychological factors, FIQ, care-management skills	Medium
Hooten et al., 2007 [42]	US	Noncontrolled study	Comparison between male and female	N/A	66 (7)	QOL, pain, psychological factors, pain interference, drug usage	Low
Hooten et al., 2007 [43]		Noncontrolled study	Pre-post evaluation	N/A	159 (17)	QOL, pain, pain interference, activity, psychological factors	Low
Kas et al., 2015 [44]	US	Controlled trial	Comparison with additional treatment	Multidisciplinary + increased exercise	79 (0)	FIQ	Medium
Keel et al., 1998 [45]	Switzerland	RCT	Comparison with control	Relaxation training	32 (5)	Pain, psychological factors, sleep, changes in drug treatment, satisfaction	Low
Kroese et al., 2009 [46]	Netherlands	Noncontrolled study	Pre-post evaluation	N/A	105 (5)	FIQ, QOL, satisfaction	Low
van Eijk-Hustings et al., 2013 [47]		RCT	Comparison with control	Aerobic exercise and TAU	203 (69)	QOL, activity, FIQ, healthcare use, sick leave	Medium
Lemstra and Olszynski, 2005 [48]	Canada	RCT	Comparison with control	Family physician	79 (8)	Pain, pain interference, psychological factors	High
Lera et al., 2009 [49]	Spain	RCT	Comparison with additional treatment	Multidisciplinary + CBT	83 (27)	QOL, FIQ, psychological factors, tender points count	High
Marcus et al., 2014 [50]	US	Noncontrolled study	Pre-post evaluation	N/A	274 (67)	Pain, ADL, movement, FIQ	Low
Martìn et al., 2012 [51]	Spain	RCT	Comparison with control	Pharmacotherapy	180 (70)	QOL, pain, psychological factors, pain interference, FIQ, satisfaction	Medium
Martìn et al., 2014 [52]		RCT	Comparison with control	Pharmacotherapy	180 (70)	FIQ, psychological factors, satisfaction	Medium
Martìn et al., 2014 [53]		RCT	Comparison with control	Pharmacotherapy	180 (70)	FIQ	Medium
Martìn et al., 2014 [54]		RCT	Comparison with control	Pharmacotherapy	180 (70)	Pain, FIQ	Medium
Martins et al., 2014 [55]	Brazil	Controlled trial	Comparison with control	TAU	27 (0)	QOL, pain, psychological factors, FIQ, sleep	Low
Mengshoel et al., 1995 [56]	Norway	Noncontrolled study	Pre-post evaluation	N/A	16 (3)	Pain, fatigue, sleep, adjustments of daily life	Low
Michalsen et al., 2013 [57]	Germany	Controlled trial	Comparison with additional treatment	Other multidisciplinary treatments	48 (6)	Pain, psychological factors, FIQ, sleep, BMI	Medium
Nielson and Jensen, 2004 [58]	Canada	Noncontrolled study	Outcome predictors	N/A	253 (55)	QOL, pain, psychological factors, pain interference, activity	Medium
Persson et al., 2012 [59]	Sweden	Noncontrolled study	Pre-post evaluation	N/A	813 (304)	Pain, psychological factors, pain interference, activity	Low
Ripley et al., 2003 [60]	US	Noncontrolled study	Pre-post evaluation	N/A	21 (0)	Pain, psychological factors, FIQ, fatigue, sleep, comorbidities	Low
Romeyke et al., 2014 [61]	Austria	Controlled trial	Comparison with control	Multidisciplinary + hyperthermia	104 (0)	Pain, chronic pain stage, severity of symptoms, pain interference	Low
Salgueiro et al., 2013 [62]	Spain	Noncontrolled study	Outcome predictors	N/A	72 (0)	Pain, FIQ, QOL, psychological factors	Low
Saral et al., 2016 [63]	Turkey	RCT	Comparison with control	Long term versus short term versus no treatment	66 (7)	QOL, pain, psychological factors, FIQ, fatigue, sleep, tender points count, pain threshold, physical functioning	Medium
Stein and Miclescu, 2013 [64]	Sweden	Noncontrolled study	Pre-post evaluation	N/A	59 (8)	Pain, psychological factors, QOL, sick leave duration, healthcare use, drug usage	Low
Suman et al., 2009 [65]	Italy	Noncontrolled study	Pre-post evaluation	N/A	25 (0)	Pain, psychological factors, tender points count, pain threshold	Low
Turk et al., 1998 [66]	US	Noncontrolled study	Pre-post evaluation	N/A	70 (3)	Pain, psychological factors, pain interference, FIQ, disability, marital satisfaction	Medium
Van Koulil et al., 2011 [67]	Netherlands	RCT	Comparison with control	WL	158 (45)	Physical fitness	High
van Wilgen et al., 2007 [68]	Netherlands	Noncontrolled study	Pre-post evaluation	N/A	65 (0)	QOL, pain, psychological factors, FIQ, physical fitness	Low
Wennemer et al., 2006 [69]	US	Noncontrolled study	Pre-post evaluation	N/A	23 (3)	QOL, FIQ, movement	Low
Worrel et al., 2001 [70]	US	Noncontrolled study	Pre-post evaluation	N/A	100 (26)	Pain, psychological factors, FIQ, pain interference, sleep, fatigue	Low
Pfeiffer et al., 2003 [71]		Noncontrolled study	Pre-post evaluation	N/A	100 (22)	QOL, FIQ, psychological factors	Low
Oh et al., 2010 [72]		Noncontrolled study	Pre-post evaluation	N/A	984 (463)	QOL, FIQ, satisfaction	Low
Vincent et al., 2013 [73]		Noncontrolled study	Pre-post evaluation	N/A	7 (0)	QOL, FIQ, psychological factors, fatigue	Low

**Table 3:** Description of the rehabilitation programs.

Reference	Approach	Format	Setting	Weeks	Hours	Professionals	Therapeutic components	Notes
Åkerblom et al., 2015 [25]	Multidisciplinary	Group	Outpatient	5	126	Psychologist, physician, physiotherapist, occupational therapist, social worker	Education, exercise, CBT, pain management techniques, relaxation, occupational therapy	Program for chronic pain rehabilitation based on a CBT framework. The group sessions are jointly conducted. The patient's caregiver is involved. Includes a follow-up intervention.

Amris et al., 2014 [26]	Interdisciplinary	Group	Outpatient	2	35	Psychologist, physician, nurse, physiotherapist, occupational therapist	Education, exercise, group discussion, pain management techniques	Scheduled team conferences, with a session jointly conducted by the psychologist and the rheumatologist.

Anderson and Winkler, 2006 [27]	Multidisciplinary	Individual	Outpatient	48	N/A	Psychologist, physician, nurse, physiotherapist, dietitian, aquatic fitness instructor	Education, land and pool exercise, auricular therapy, microcurrent therapy, CBT, massage, nutritional counseling	Program based on three phases with different treatment intensities. The various components are juxtaposed.

Angst et al., 2006 [28] Angst et al., 2009 [29]	Interdisciplinary	Both	Inpatient	4	100	Psychologist, physician, nurse, physiotherapist, occupational therapist, Qi-gong instructor, creative and humor therapists	Education, exercise, group discussion, CBT, pain management techniques, Qi-gong, humor therapy	Based on the integration of traditional and Chinese medicine, exercise and physiotherapy, and different psychotherapeutic methods. The team received education on interdisciplinary work and participates in weekly meetings.

Bailey et al., 1999 [30]	Interdisciplinary	Group	Outpatient	12	72	Physiotherapist, occupational therapist, social worker. The team is supported by pharmacist, dietitian, kinesiologist	Education, exercise, discussion, pain management techniques	Based on exercise and education, performed by an interdisciplinary group and a supporting group; patients can access the members of the teams.

Bourgault et al., 2015 [31]	Interdisciplinary	Group	Outpatient	11	22,5	Physician, facilitator expert in the psychological area and facilitator expert in the physical area	Education, exercise, group discussion, pain management techniques based on CBT	Based on interactive sessions jointly conducted by the two operators, who are described as “facilitators.” The exercises and psychological components are tailored to the single patient.

Carbonell-Baeza et al., 2011 [32] Carbonell-Baeza et al., 2011 [33]	Multidisciplinary	Group	Outpatient	12	45	Psychologist, physiotherapist, fitness specialist	Education, exercise, Acceptance and Commitment Therapy, pain management techniques, massage	The program is based on exercise and education; the exercise sessions are supervised by both a physiotherapist and a fitness specialist.
Carbonell-Baeza et al., 2012 [34]				16	60			

Casanueva-Fernández et al., 2012 [35]	Multidisciplinary	Individual	Outpatient	8	8	The team is not described	Education, exercise, ischemic pressure, massage, thermal therapy	The medical treatment is associated with a program of education and physical treatments.

Castel et al., 2013 [36]	Multidisciplinary	Group	Outpatient	12	48	Psychologist, physiotherapist	Exercise, CBT	The pharmacologic treatment is accompanied by CBT and physical therapy.

Cedraschi et al., 2004 [37]	Multidisciplinary	Group	Outpatient	6	18	Physician, physiotherapist, occupational therapist	Education, pool and land exercise, group discussion, pain management techniques	The program is based on the patient's self-management of his/her symptoms. The educational sessions are conducted by the whole multidisciplinary team.

De Rooij et al., 2013 [38]	Multidisciplinary	Both	Outpatient	7	49	Psychologist, physician, physiotherapist, occupational therapist, social worker	Education, exercise, CBT, pain management techniques	The program is tailored to the patient's goals and regular meetings are scheduled to facilitate team discussions.

Gustafsson et al., 2002 [39]	Multidisciplinary	Group	Outpatient	12	104	Physician, nurse, physiotherapist, occupational therapist, social worker	Education, exercise, group discussion, pain management techniques	The education component is jointly administered by the nurse and the physiotherapist. At the end of the program, the team members meet at a conference the patient, his employer, and the local insurance officer to discuss the treatment plan.

Hammond and Freeman, 2006 [40]	Education-exercise program	Group	Outpatient	10	20	Physiotherapist, occupational therapist	Education, exercise	Education is also based on self-management. The program is administered by either the physiotherapist or the occupational therapist, whereas the other one acts as a supervisor.

Hamnes et al., 2012 [41]	Multidisciplinary	Group	Inpatient	1	70	Physician, nurse, physiotherapist, occupational therapist, social worker, dietitian	Education, exercise, group discussion, pain management techniques based on cognitive-behavioral principles, nutritional counseling	Complex and intensive treatment based on self-management, with involvement of the patient's partner. The different therapeutic components are juxtaposed.

Hooten et al., 2007 [42] Hooten et al., 2007 [43]	Multidisciplinary	Both	Outpatient	3	150	Psychologist, mental health therapist, physician, physiotherapist, occupational therapist, nurse, chemical dependency counselors	Education, exercise, CBT, pain management techniques based on a cognitive-behavioral framework, biofeedback, relaxation, opioid discontinuation	Description of the treatment found in the research by Townsend et al., 2008 [74]. The team includes a large number of operators from various disciplines. Gradual withdrawal from opioids under the supervision of the physician.

Kas et al., 2015 [44]	Multidisciplinary	Individual	Outpatient	10	25–40	Psychologist, physician, physiotherapist, occupational therapist	Aerobic and strengthening exercise, CBT/Acceptance and Commitment Therapy, biofeedback, occupational therapy	The study evaluates the effects of adding strength exercises to an existing multidisciplinary treatment. The various components are juxtaposed.

Keel et al., 1998 [45]	Integrated treatment	Group	Outpatient	15	30	Physiotherapist, psychologist, psychiatrist	Exercise, education, pain management strategies, relaxation, discussion	Integrated psychological group treatment based on self-management. The sessions are jointly led by the operators.

Kroese et al., 2009 [46]	Multidisciplinary	Group	Outpatient	12	108	Psychologist, physiotherapist, occupational therapist	Education, exercise, group discussion, pain management techniques, sociotherapy, creative arts therapy	Physicians are not involved in order to prevent the medicalization of the syndrome. Five aftercare meetings are scheduled to prevent relapses. Based on the patient's needs, more intensive therapeutics could be added.
van Eijk-Hustings et al., 2013 [47]				12 + aftercare	108			

Lemstra and Olszynski, 2005 [48]	Multidisciplinary	Group	Outpatient	6	24	Physician, psychologist, physiotherapist, massage therapist, dietitian	Exercise, pain management techniques, education, massage	The program includes rheumatologist and physical therapist intake, group exercise and education, individual massage therapy sessions, and rheumatologist and physical therapist discharge.

Lera et al., 2009 [49]	Multidisciplinary	Group	Outpatient	16	14	Physician, physiotherapist, rehabilitation practitioner, additional group: psychologist	Education, exercise, additional group: CBT	Supported by an individual medical treatment, four of the fourteen sessions are jointly conducted by the rheumatologist and a rehabilitation practitioner.

Marcus et al., 2014 [50]	Multidisciplinary	Group	Outpatient	6	36–48	Psychologist, physician, physiotherapist, occupational therapist	Education, exercise, CBT, pain management techniques	Mainly based on education, exercise, and the development of pain management skills.

Martìn et al., 2012 [51] Martìn et al., 2014 [52] Martìn et al., 2014 [53] Martìn et al., 2014 [54]	Interdisciplinary	Group	Outpatient	6	21	Psychologist, physician, physiotherapist	Education, exercise, CBT, pain management techniques, massage	The psychological, medical, educational, and physiotherapeutic approaches are coordinated; however, there is no information about this process.

Martins et al., 2014 [55]	Interdisciplinary	Group	Outpatient	12	12	Psychologist, physician, physiotherapist, occupational therapist, social worker	Education, exercise, pain management techniques based on cognitive-behavioral principles	It is unclear how the operators are integrated.

Mengshoel et al., 1995 [56]	Multidisciplinary	Group	Outpatient	10	20	Physician, physiotherapist, dietitian	Education, exercise, group discussion	Based on lessons and exercise sessions jointly conducted by two team members.

Michalsen et al., 2013 [57]	Multidisciplinary	Group	Inpatient	2	N/A	Psychologist, physician, physiotherapist	Land and pool exercise, thermal therapy, CBT, education, additional group: fasting and nutritional therapy	Intensive programs; the components are juxtaposed.

Nielson and Jensen, 2004 [58]	Multidisciplinary	Both	Outpatient	4	N/A	Psychologist, physician, physiotherapist, occupational therapist	Education, exercise, CBT, occupational therapy, opioid tapering	Aimed at improving pain management skills and physical and psychological functioning, based on a cognitive-behavioral model.

Persson et al., 2012 [59]	Interdisciplinary	Both	Outpatient	5	150	Psychologist, physician, nurse, physiotherapist, occupational therapist, social worker	Education based on cognitive-behavioral principles, exercise, group discussion, biofeedback, pain management techniques	Based on teamwork, each operator is educated about the tools of all the disciplines and about cognitive-behavioral techniques. Includes team-based lectures; the operators participate in weekly meetings. The treatment is tailored to the patient during the first week. In the first and last weeks, individual sessions are scheduled and a final meeting with the patient, his/her caregiver, his/her employer, and the local insurance officer is planned. The team visits the patient's workplace and his/her caregiver is involved. Follow-up meeting two months after discharge.

Ripley et al., 2003 [60]	Multidisciplinary	Individual	Outpatient	24	N/A	Psychologist, physician, nurse, occupational therapist	Education, dietary modification, pain management techniques, nutraceuticals	Initial screening made by a physician with a social worker who evaluates with the family whether the patient is able and willing to complete the program and recommends if needed to add to the treatment also psychological counseling, group sessions, or other strategies. The treatment is tailored to the patient's needs and based on the response to the different therapeutic components.

Romeyke et al., 2014 [61]	Interdisciplinary	Group	Inpatient	2	N/A	Psychologist, physician, nurse, physiotherapist, naturopathy specialist, ergonomic specialist	Hyperthermia, exercise, CBT, pain management techniques, massage	The nurses supervise the treatment and act as process coordinators. Weekly team meetings are scheduled.

Salgueiro et al., 2013 [62]	Multidisciplinary	Both	Outpatient	4	60	Psychologist, physician, nurse, occupational therapist	Pharmacotherapy, CBT, exercise, pain management techniques, occupational therapy, education	Complex intervention including pharmacotherapy, CBT, physical and occupational therapy, and education, with focus on tackling the patient's misconception about the syndrome.

Saral et al., 2016 [63]	Interdisciplinary	Group	Outpatient	10	46	Psychologist, physician, physiotherapist	Education, exercise, CBT	The long-term program includes a full day of education led by the investigators and then a group CBT along with a program of physical therapy with prescription of home exercises. The short-term program includes the same therapeutic components administered in two days.

Stein and Miclescu, 2013 [64]	Multidisciplinary	Group	Outpatient	6	60	Psychologist, physician, physiotherapist, occupational therapist	Education, exercise, CBT	Interdisciplinary assessment and discussion during the intake; at the end, two or three operators in representation of the whole team meet the patient and his/her family members. There is the possibility for the patient to individually contact the operators. The patient meets again the whole team at the end of the treatment; his/her employer and other key figures are invited.

Suman et al., 2009 [65]	Multidisciplinary	Individual	Inpatient	3	75	Psychologist, physician, physiotherapist	Education, exercise, CBT	Academic setting; at each weekend, the patient returns to his/her home and tries to practice pain management skills. The treatment components are juxtaposed.

Turk et al., 1998 [66]	Interdisciplinary	Group	Outpatient	4	24	Psychologist, physician, physiotherapist, occupational therapist	Education, exercise, CBT, pain management techniques	Each half-day session includes medical, physical, psychologic, and occupational components. Team meetings are planned; the treatment is supervised by a rheumatologist.

Van Koulil et al., 2011 [67]	Multidisciplinary	Group	Outpatient	8	32	Psychologist, physiotherapist, social worker	Exercise, CBT, pain management techniques	The treatment is mainly based on CBT and exercise; the intervention is tailored to the patient.

van Wilgen et al., 2007 [68]	Multidisciplinary	Group	Outpatient	17	N/A	Physiotherapist, occupational therapist, social worker, trainer	Education, exercise, relaxation	Mainly based on education and physical therapy, provided by trainers and physical therapists.

Wennemer et al., 2006 [69]	Multidisciplinary	Group	Outpatient	8	48	Physician, physiotherapist, occupational therapist	Education, Feldenkrais or Tai-Chi exercise, relaxation, occupational therapy	Progressive program incorporating education, exercise, and stress reduction techniques.

Worrel et al., 2001 [70] Pfeiffer et al., 2003 [71] Oh et al., 2010 [72]	Interdisciplinary	Group	Outpatient	<1	8	Physician, nurse, physical and occupational therapist	Education, exercise, group discussion, pain management techniques	During the first day, the nurse is responsible for the assessment phase, leads the first education session, and facilitates a group discussion. The day after, physical and occupational therapists complete the program with lectures and exercises. Other professionals are involved if necessary. The participation of the patient's family is encouraged.
Vincent et al., 2013 [73]				1	42	Psychologist, physician, nurse, physiotherapist, occupational therapist	Patient education, family education, pain management techniques	Modified version of the FTP treatment. The program is implemented in one week and includes the same components plus planned involvement of the family members and follow-up contacts with the nurse.
